# Fedratinib reveals chemotherapeutic potential in esophageal squamous cell cancer

**DOI:** 10.3389/fphar.2025.1689663

**Published:** 2025-12-16

**Authors:** Man Luo, Jiang Zhu, Yong Yang, Chongbo Liao, Xiao Liu, Maoju Tang, Lei Xu, Xiaowu Zhong, Qiang Ma, Xiaolan Guo

**Affiliations:** 1 Department of Clinical Laboratory, Affiliated Hospital of North Sichuan Medical College, Nanchong, China; 2 School of Laboratory Medicine & Translational Medicine Research Center, North Sichuan Medical College, Nanchong, China; 3 Xichang Medical College, Xichang, China

**Keywords:** fedratinib, esophageal squamous cell cancer, JAK2/STAT3 pathway, organoid, drug repurposing

## Abstract

**Background:**

Drug repurposing has emerged as a promising approach for discovering novel anticancer therapeutics. In this study, we systematically investigated the antitumor potential of fedratinib, a JAK2 inhibitor approved for myelofibrosis, against esophageal squamous cell carcinoma (ESCC) using integrated *in vitro*, *in vivo*, and patient-derived organoid (PDO) models. We further explored its underlying mechanisms of action.

**Methods:**

ESCC cell lines (Eca109 and KYSE150) were treated with fedratinib to evaluate its effects on proliferation, migration, apoptosis, and cell cycle distribution. Molecular changes were examined using RT-qPCR and Western blot analyses. Antitumor efficacy was further validated in subcutaneous xenograft models and ESCC PDOs. Mechanistic investigations included STAT3 overexpression and functional rescue experiments.

**Results:**

Fedratinib significantly inhibited ESCC cell proliferation and migration and induced cell cycle arrest at the G2/M phase while promoting apoptosis *in vitro*. It also suppressed tumor growth in xenograft models and showed consistent efficacy in PDOs. Mechanistically, fedratinib inhibited the activation of the JAK2/STAT3 signaling pathway and downregulated the expression of vimentin, survivin, and cyclin D1. Overexpression of STAT3 reversed these molecular alterations and diminished the functional effects of fedratinib. Similarly, ectopic expression of survivin, vimentin, or cyclin D1 partially rescued the phenotypic changes induced by fedratinib.

**Conclusion:**

Fedratinib exerts antitumor effects against ESCC *in vitro*, *in vivo*, and in patient-derived organoid models by suppressing the JAK2–STAT3 signaling axis and its downstream effectors, vimentin, survivin, and cyclin D1.

## 1 Introduction

Esophageal cancer (EC) is a highly aggressive malignancy and a major global health burden. In 2020, approximately 604,000 new cases and 544,000 deaths were reported worldwide, making EC the seventh most diagnosed cancer and the sixth leading cause of cancer-related mortality (Sung et al., 2021). China bears a disproportionate share of this disease, accounting for more than half of all global EC cases, with esophageal squamous cell carcinoma (ESCC) representing the predominant histological subtype (Zhu et al., 2023). Notable epidemiological disparities exist across the country, as incidence rates in rural central China are approximately four times higher than those in urban northeastern regions, underscoring the influence of geographic and socioeconomic factors on ESCC distribution (Wang et al., 2021). Current clinical management of ESCC includes endoscopic therapy, surgery, radiotherapy, and chemotherapy (Rogers et al., 2022). However, treatment outcomes remain unsatisfactory, and the five-year survival rate for ESCC patients remains low (Waters and Reznik, 2022). Therefore, the discovery of novel and effective therapeutic agents for ESCC is of urgent clinical importance.

Drug repurposing has emerged as an attractive and efficient strategy to accelerate anticancer drug development and is increasingly playing a pivotal role in the identification of novel therapeutic options across a range of malignancies (Schipper et al., 2022; Xia et al., 2024). Although drug repurposing efforts are often initially evidence-based in cellular and animal models (Witham et al., 2023), there remains a lack of comprehensive safety data regarding potential adverse effects when these agents are applied to new disease contexts (Shrestha et al., 2023). Therefore, rigorous preclinical evaluation remains essential. In this regard, patient-derived cancer organoids (PDOs) have demonstrated a high degree of histological and molecular fidelity to the original tumors, and are now widely regarded as a robust *in vitro* platform for assessing drug efficacy (Yuan et al., 2023).

Fedratinib is an orally administered, selective JAK2 tyrosine kinase inhibitor approved by the FDA (McLornan et al., 2021). It received its first global approval in the United States in August 2019 for the treatment of adult patients with intermediate-2 or high-risk primary or secondary myelofibrosis (Blair, 2019). Previous studies have indicated the broader potential of fedratinib. For instance, fedratinib-based combination therapy can effectively induce cytotoxicity in KBV20C cancer cells overexpressing the drug-efflux pump P-glycoprotein (Oh et al., 2022). Moreover, pharmacogenomic analyses suggest that fedratinib may exhibit utility in head and neck squamous cell carcinoma (HNSCC) exhibiting low KRT18 expression (Gu et al., 2022). Despite these insights, the therapeutic potential of fedratinib in solid tumors has not been systematically investigated.

In this study, we identify fedratinib—an FDA-approved JAK2 inhibitor—as a promising repurposed candidate for ESCC treatment. Using a comprehensive approach that integrates *in vitro*, *in vivo*, and patient-derived organoid models, we demonstrate that fedratinib potently inhibits ESCC proliferation and migration. Although the JAK2/STAT3 pathway has been implicated in various cancers, the systematic evaluation of fedratinib (a selective JAK2 inhibitor) in ESCC, particularly using a combination of cell lines, xenograft models, and patient-derived organoids (PDOs) to validate its efficacy and mechanism, has not been previously reported. Mechanistically, fedratinib acts by suppressing the JAK2/STAT3 signaling pathway, resulting in the downregulation of vimentin, survivin, and cyclin D1. Our results not only underscore the translational potential of fedratinib in ESCC but also provide a molecular foundation for its further clinical development.

## 2 Materials and methods

### 2.1 Reagents and antibodies

Fedratinib was obtained from TOPSCIENCE (Shanghai, China). Cell culture media (RPMI-1640) and fetal bovine serum (FBS) were purchased from Gibco (Grand Island, NY, United States). The CCK-8 assay kit was supplied by Beyotime Biotechnology (Shanghai, China). The pCMV-STAT3 plasmid was acquired from HedgehogBio (Shanghai, China), and the FITC Annexin V Apoptosis Detection Kit was purchased from BD Biosciences (Franklin Lakes, NJ, United States).

Primary antibodies against JAK2, p-JAK2, STAT3, and p-STAT3 were purchased from Abcam (Cambridge, MA, United States). Antibodies targeting vimentin and survivin were procured from Cell Signaling Technology (Danvers, MA, United States). Additional primary antibodies, including those for GAPDH, β-actin, caspase-3, cleaved caspase-3, PARP, cleaved PARP, cyclin B1, cyclin D1, and CDK1, were obtained from HUABIO (Shandong, China). Corresponding secondary antibodies (goat anti-rabbit and goat anti-mouse) were sourced from BOSTER (Wuhan, China).

### 2.2 Cell culture

The ESCC cell lines Eca109 and KYSE150 were acquired from the Translational Medicine Research Center at North Sichuan Medical College. Cells were maintained in RPMI-1640 medium supplemented with 10% fetal bovine serum and incubated at 37 °C in a humidified atmosphere containing 5% CO_2_.

### 2.3 Initial drug screening

A high-throughput screening of 320 FDA-approved drugs was performed in ESCC cell lines. Cells were treated with each drug at a concentration of 10 μM for 48 h. DMSO was used as a vehicle control. Cytotoxicity was assessed using CCK-8 assays, and hits were identified based on a predefined threshold of >50% reduction in cell viability compared to controls.

### 2.4 Cell viability measurement

Eca109 and KYSE150 were seeded into 96-well plates at a density of 8 × 10^3^ cells per well in RPMI-1640 medium containing 10% FBS. After 12 h, the cells were treated with either DMSO or various concentrations of fedratinib for 48 h; alternatively, they were exposed to fedratinib at 3 or 6 μM for different time intervals. Following treatment, 10 μL of CCK-8 reagent was added to each well, and the plates were incubated at 37 °C under 5% CO_2_ for 2 h. Absorbance at 450 nm was measured using a Sunrise™ microplate reader (TECAN, NC, United States). All experiments were performed in triplicate.

### 2.5 Plate colony formation assay

Eca109 and KYSE150 cells were seeded into 12-well plates at a density of 5 × 10^2^ cells per well and allowed to adhere. After 5 days, the culture medium was replaced with 2 mL of fresh RPMI-1640 containing 10% FBS and supplemented with either DMSO or fedratinib (3 and 6 μM). Treatment continued until distinct cell clones became visible macroscopically. The cells were then fixed with methanol for 10 min and stained with crystal violet solution for an additional 10 min. Stained colonies were imaged using a GT-S650 scanner (Epson).

### 2.6 Transwell assay

Cells in the logarithmic growth phase were seeded into 6-well plates at a density of 5 × 10^5^ cells per well. After 24 h, the cells were treated with DMSO or fedratinib (3 and 6 μM) for 48 h. Following treatment, the cells were harvested by trypsinization using 0.25% trypsin, resuspended in serum-free medium, and adjusted to a density of 5 × 10^5^ cells/mL. Then, 200 μL of the cell suspension was added to the upper chamber of a Transwell insert, whereas the lower chamber was filled with 500 μL of RPMI-1640 medium containing 10% FBS as a chemoattractant. After 24 h of incubation, the cells remaining on the upper surface of the membrane were carefully removed. The cells that had migrated to the lower surface were fixed with methanol, stained with crystal violet, and imaged under a microscope.

### 2.7 Scratch assay

Cells were seeded into 6-well plates at a density of 5 × 10^5^ cells per well and cultured until reaching approximately 90% confluence. A uniform wound was then created in each well by scratching the cell monolayer with a sterile 10-μL pipette tip. After being gently washed twice with PBS to remove detached cells, the cells were maintained in RPMI-1640 medium containing 1% FBS and treated with either DMSO or fedratinib (3 and 6 μM). Wound closure was monitored and photographed at designated time points over a 24-hour incubation period.

### 2.8 RNA extraction and real-time PCR

Total RNA was extracted using TRIzol reagent according to the manufacturer’s protocol. Subsequently, 1 μg of RNA was reverse-transcribed into cDNA using a commercial reverse transcription system kit. Quantitative real-time PCR was performed on a Roche LightCycler® 96 system (Roche, United States). Each 10 μL reaction mixture contained 5 μL of 2× TB Green Premix Ex Taq II (Takara), 0.2 μL of forward primer (10 μM, Sangon, China), 0.2 μL of reverse primer (10 μM, Sangon, China), 0.5 μL of cDNA template, and 4.1 μL of ddH_2_O. The thermal cycling conditions were as follows: initial denaturation at 95 °C for 30 s, followed by 35 cycles of 95 °C for 5 s, 58 °C for 30 s, and 72 °C for 20 s. All reactions were performed in triplicate. The relative mRNA expression levels of vimentin, survivin, and cyclin D1 were calculated using the 2^(-ΔΔCt) method, with β-actin serving as the internal control. The primer sequences used were as follows: vimentin: forward: 5′-GGA​CCA​GCT​AAC​CAA​CGA​CA-3′, reverse: 5′-AAG​GTC​AAG​ACG​TGC​CAG​AG-3′; cyclin D1: forward: 5′-GAT​GCC​AAC​CTC​CTC​AAC​GA-3′, reverse: 5′-GGA​AGC​GGT​CCA​GGT​AGT​TC-3′; survivin: forward: 5′-CAG​CCC​TTT​CTC​AAG​GAC​CA-3′, reverse: 5′-TTT​CCT​TTG​CAT​GGG​GTC​GT-3′; *β-actin:* forward: 5′-CAT​GTA​CGT​TGC​TAT​CCA​GGC-3′, reverse: 5′-CTC​CTT​AAT​GTC​ACG​CAC​GAT-3′.

### 2.9 Western blotting

Cells were lysed using RIPA buffer supplemented with protease and phosphatase inhibitors. Protein concentrations were quantified, and 40 μg of total protein per sample was separated using SDS-PAGE and subsequently transferred onto a polyvinylidene difluoride (PVDF) membrane. The membranes were blocked with 5% skim milk for 1 h at room temperature and then incubated with primary antibodies overnight at 4 °C. After washing, the membranes were probed with appropriate secondary antibodies for 1 h at room temperature. Protein bands were visualized using an enhanced chemiluminescence (ECL) detection system (Vilber Fusion FX7, France).

### 2.10 Plasmid transfection

Cells were seeded in 6-well plates at a density of 3 × 10^5^ cells per well and cultured for 24 h. The cells were then transfected with either pcDNA3.1–3×HA-STAT3 or pCMV-STAT3 plasmids using Lipofectamine 2000 reagent in OPTI-MEM medium. After 6 h, the transfection medium was replaced with fresh DMEM supplemented with 5% FBS, and the cells were further incubated for 48 h.

### 2.11 Tumor-bearing nude mice model construction and treatment

Four-week-old male BALB/c nude mice were obtained from the Experimental Animal Center of North Sichuan Medical College. All animal procedures were approved by the Institutional Animal Care and Use Committee of North Sichuan Medical College. Mice were housed under standard conditions (temperature: 25 °C; humidity: 55%; 12 h/12 h light/dark cycle) at the center. KYSE150 cells (1 × 10^6^) were subcutaneously implanted into the dorsal flank of each mouse. The mice were then randomly divided into two groups and received intraperitoneal injections of either DMSO or fedratinib once every 2 days. The dose of 25 mg/kg every 2 days was selected based on previous pharmacokinetic and efficacy studies of fedratinib in mouse models, and its conversion to a human equivalent dose falls within the clinically achievable and tolerated range reported in human trials. Tumor length and width were measured every 2 days using a vernier caliper, and tumor volume was calculated using the following formula: volume (mm^3^) = length × width^2^/2. On day 19, the mice were euthanized, and lung, liver, spleen, kidney, heart, and tumor tissues were collected. The tissues were fixed in 4% paraformaldehyde, embedded in paraffin, and sectioned for hematoxylin and eosin (H&E) staining and immunohistochemical analysis.

### 2.12 Immunohistochemical staining

Paraffin-embedded sections from esophageal cancer xenografts were subjected to immunohistochemical staining. In brief, after deparaffinization and rehydration, endogenous peroxidase activity was quenched by incubation with 0.3% hydrogen peroxide at 37 °C for 30 min. Antigen retrieval was then performed using citrate buffer. Subsequently, the sections were blocked with 5% bovine serum albumin (BSA) for 30 min at 37 °C. The sections were incubated with primary antibody overnight at 4 °C, followed by incubation with a corresponding secondary antibody at 37 °C for 30 min. Color development was carried out using DAB as the chromogen, and the nuclei were counterstained with hematoxylin. Finally, the tissue sections were dehydrated, cleared, mounted, and imaged under a microscope.

### 2.13 HE stains of tissue specimens

Deparaffinized and rehydrated tissue sections were stained with hematoxylin for 5 min, followed by differentiation in 1% acid alcohol. The sections were then counterstained with eosin for 3 min. Finally, the sections were dehydrated through a graded ethanol series, cleared in xylene, and mounted with neutral resin for microscopic examination.

### 2.14 ESCC organoids and imaging-based drug response analysis

ESCC tissues were dissociated, and the resulting tumor cells were resuspended in organoid culture medium. The cell suspension was mixed with growth factor-reduced Matrigel (Corning) at a 1:1.8 volume ratio, and 20 µL aliquots of the mixture were plated as 5–7 drops per well in 6-well plates. Matrigel was allowed to polymerize at 37 °C, after which an organoid medium was added to cover the gel droplets. Cultures were maintained at 37 °C in a humidified 5% CO_2_ incubator and passaged every 3–4 weeks by manual dissociation and re-embedding in fresh Matrigel at a 1:3 to 1:4 split ratio.

For drug testing, cell–Matrigel mixtures containing 8 × 10^4^ cells in 10 µL were plated as central droplets in 96-well plates. After gel formation, culture medium was added, and the organoids were cultured for 7 days. Subsequently, the organoids were treated with fedratinib or DMSO for 72 h. Caspase-3/7 activation was monitored in real time over 72 h using an IncuCyte® live-cell imaging system. Cell viability was quantified using the CellTiter-Glo® luminescent ATP assay. Dose–response curves were generated using nonlinear least squares regression (variable slope, four parameters), and the half-maximal inhibitory concentration (IC_50_), maximal inhibitory effect (E_max), and Hill slope (HS) were calculated using GraphPad Prism 9.

### 2.15 Statistical analysis

Data are presented as the mean ± standard deviation (SD). Differences between groups were assessed using Student’s t-test or one-way analysis of variance (ANOVA), as appropriate. A *p*-value of less than 0.05 was considered statistically significant.

## 3 Results

### 3.1 Fedratinib inhibits the proliferation and migration of ESCC

**FIGURE 1:**
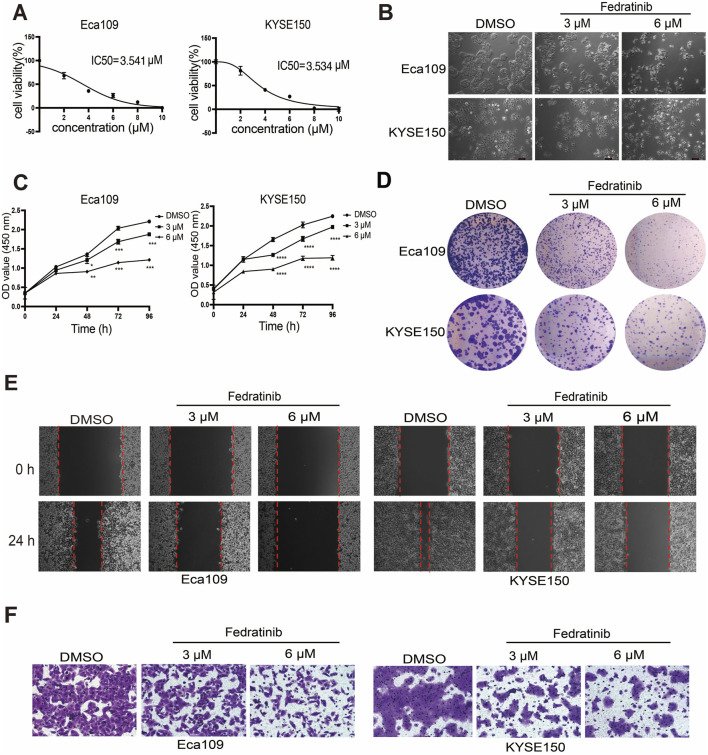
Fedratinib inhibits proliferation and migration of ESCC cells. **(A)** Viability of Eca109 and KYSE150 cells treated with the indicated concentrations of fedratinib (0–10 μM) for 48 h, assessed using the CCK-8 assay. **(B)** Morphological changes in Eca109 and KYSE150 cells after treatment with fedratinib (3 or 6 μM) or DMSO. **(C)** OD values measured at 450 nm for Eca109 and KYSE150 cells treated with fedratinib at the indicated doses and time points. **(D)** Clonogenic capacity assessed by colony formation assay in Eca109 and KYSE150 cells treated with fedratinib (3 or 6 μM) or DMSO. **(E,F)** Migration ability evaluated using the scratch assay **(E)** and Transwell assay **(F)** in Eca109 and KYSE150 cells treated with fedratinib (3 or 6 μM) or DMSO (*p < 0.05, **p < 0.01, ***p < 0.001, and ****p < 0.0001).

A high-throughput screening of 320 FDA-approved drugs (10 μM, 48 h) in ESCC cell lines identified four candidate compounds with cytotoxic potential, based on predefined optical density thresholds. Subsequent dose–response profiling excluded two candidates that failed to achieve 50% cytotoxicity across the tested concentration range (Supplementary Figure S1). Fedratinib, a selective JAK2 inhibitor, emerged as the most potent agent, with IC_50_ values of 3.541 μM in Eca109 and 3.534 μM in KYSE150 cells at 48 h (Figure 1A). Based on these results, fedratinib was tested at concentrations of 3 μM (low) and 6 μM (high) in subsequent experiments. Fedratinib induced dose-dependent morphological shrinkage in both cell lines (Figure 1B) and significantly suppressed cell viability in a time- and concentration-dependent manner (Figure 1C). Clonogenic assays further demonstrated progressive inhibition of colony formation with increasing fedratinib concentrations (Figure 1D). Moreover, wound healing and Transwell assays showed that fedratinib markedly attenuated the migratory capacity of ESCC cells compared to DMSO-treated controls (Figures 1E,F; Supplementary Figure S2A,B). Together, these findings establish fedratinib as a potent inhibitor of ESCC proliferation and migration.

### 3.2 Fedratinib induces G2/M phase arrest and apoptosis in ESCC cells

**FIGURE 2:**
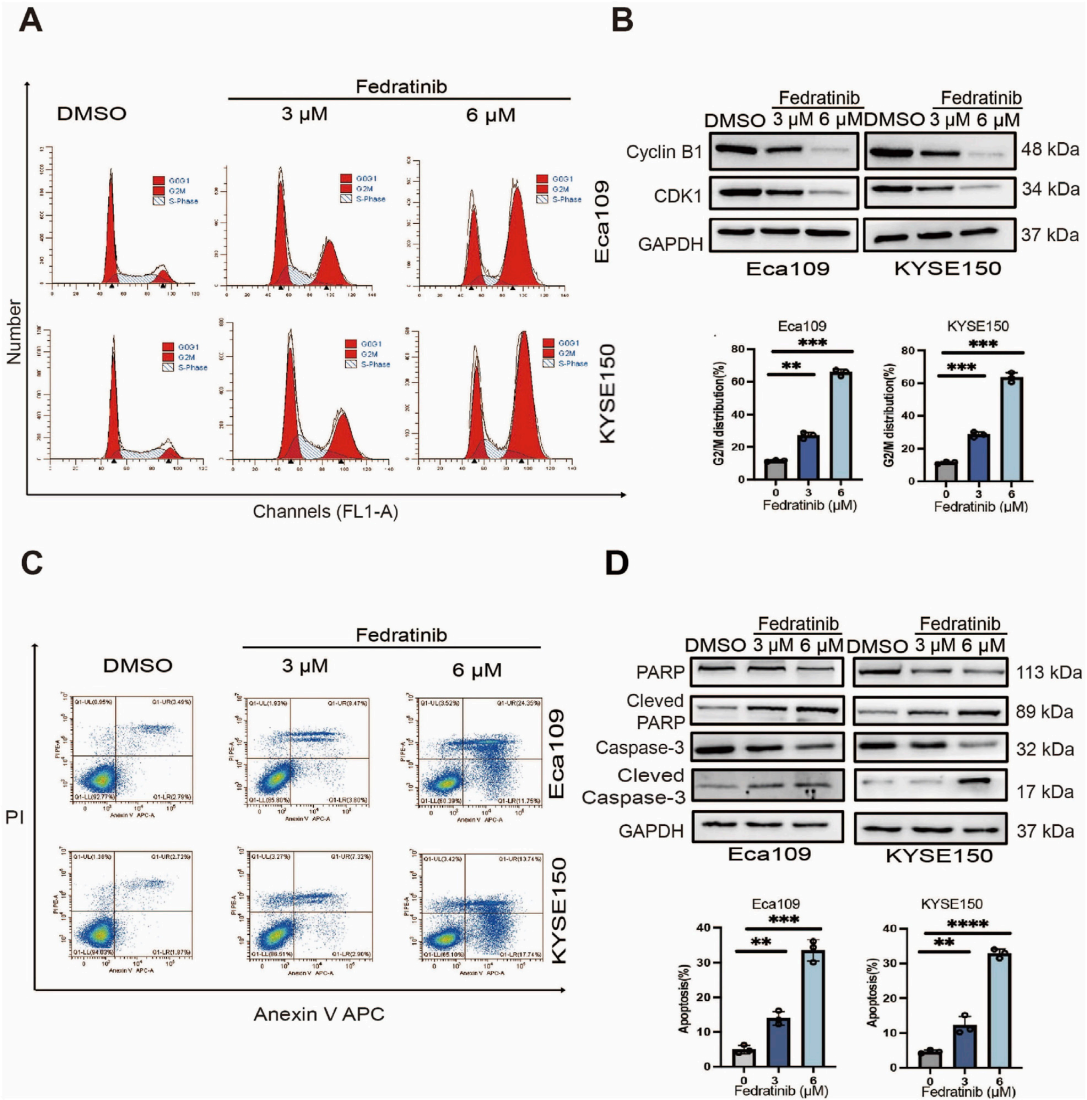
Fedratinib induces G2/M phase arrest and apoptosis in ESCC cells. **(A)** Cell cycle distribution analyzed by flow cytometry in Eca109 and KYSE150 cells treated with fedratinib (3 or 6 μM) or DMSO. **(B)** Protein levels of cyclin D1 and CDK1 detected using Western blot. **(C)** Apoptosis rate evaluated by flow cytometry in fedratinib-treated Eca109 and KYSE150 cells. **(D)** Expression of PARP, cleaved PARP, caspase-3, and cleaved caspase-3 analyzed using Western blot (**p < 0.01, ***p < 0.001, and ****p < 0.0001).

Cell cycle dysregulation is a hallmark of cancer proliferation (Schuhwerk and Brabletz, 2023). To determine whether fedratinib influences cell cycle progression in ESCC, Eca109 and KYSE150 cells were treated with graded concentrations of fedratinib and subjected to PI staining and flow cytometric analysis. The results showed that fedratinib significantly increased the proportion of cells in the G2/M phase compared to DMSO-treated controls (Figure 2A; Supplementary Figure S3A). Consistent with the observed G2/M accumulation, immunoblot analysis showed a dose-dependent downregulation of key G2/M regulatory proteins, including Cyclin B1 and CDK1, in both cell lines (Figure 2B), indicating that fedratinib induces G2/M phase arrest.

Given that apoptosis represents a major mechanism underlying the antitumor activity of many chemotherapeutic agents (Morana et al., 2022), we next evaluated the proapoptotic potential of fedratinib. Annexin V-FITC/PI staining demonstrated a significant increase in the percentage of apoptotic cells upon fedratinib treatment (Figure 2C; Supplementary Figure S3B). This result was further supported by the marked upregulation of cleaved Caspase-3 and cleaved PARP, key executioners of apoptosis, as shown by Western blot (Figure 2D). Taken together, these data indicate that fedratinib suppresses ESCC proliferation by inducing G2/M phase cell cycle arrest and promoting apoptosis.

### 3.3 Fedratinib suppresses ESCC progression by inhibiting the JAK2/STAT3 pathway

**FIGURE 3:**
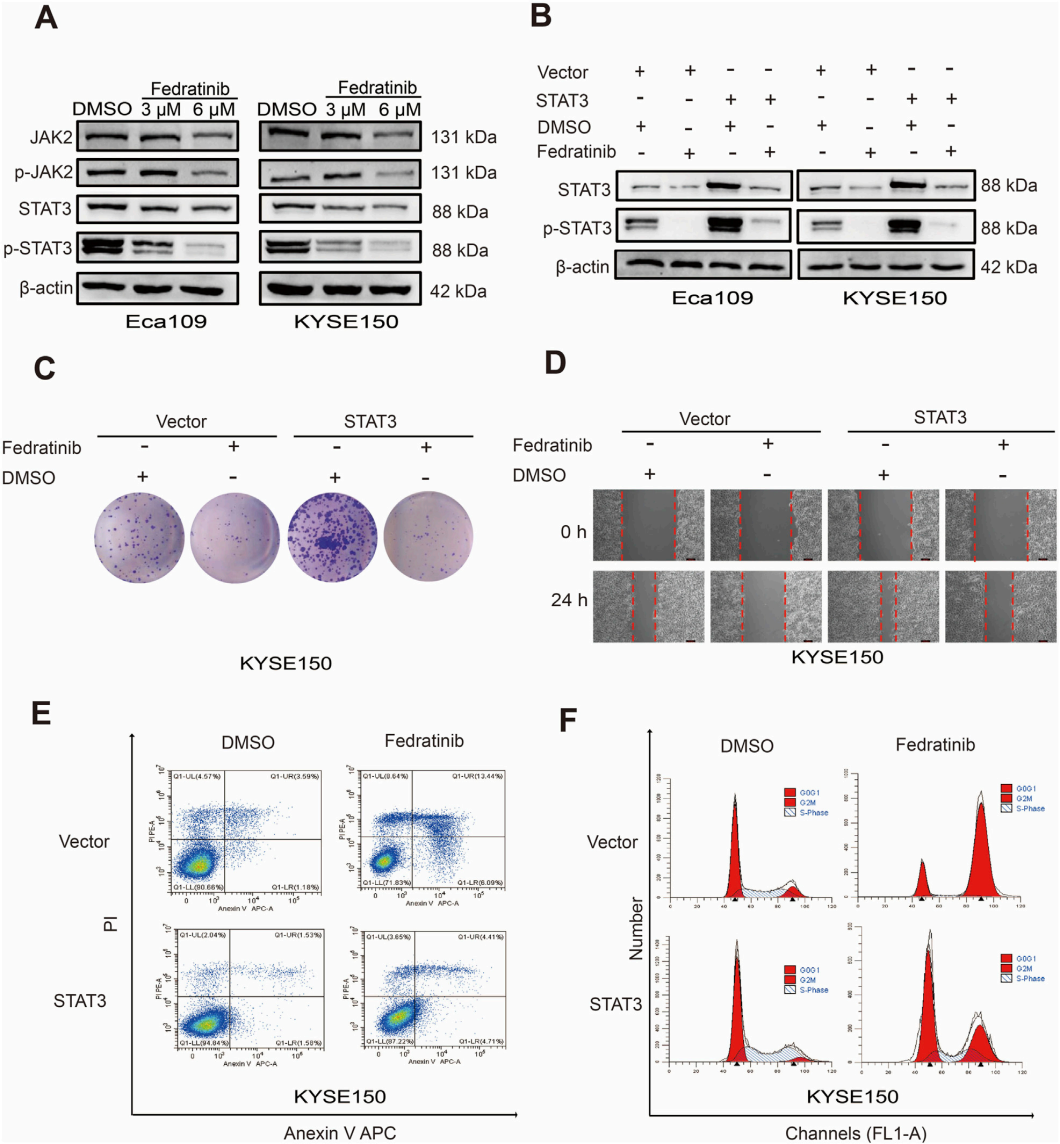
Fedratinib suppresses the JAK2/STAT3 signaling pathway in ESCC cells. **(A)** Protein expression of key components in the JAK2/STAT3 pathway in Eca109 and KYSE150 cells treated with fedratinib (3 or 6 μM) or DMSO. **(B)** STAT3 and p-STAT3 levels detected by Western blot in Eca109 and KYSE150 cells overexpressing STAT3 and treated with fedratinib. **(C)** Effect of STAT3 overexpression on proliferation assessed using the colony formation assay in KYSE150 cells treated with fedratinib. **(D–F)** Migration **(D)**, cell cycle distribution **(E)**, and apoptosis **(F)** evaluated in KYSE150 cells overexpressing STAT3 and treated with fedratinib.

As a selective JAK2 inhibitor, fedratinib is hypothesized to exert its antitumor effects in ESCC by suppressing the JAK2/STAT3 signaling pathway. Western blot analysis confirmed that treatment with fedratinib for 48 h significantly reduced the protein levels of JAK2, p-JAK2, STAT3, and p-STAT3 in both Eca109 and KYSE150 cells, with phospho-STAT3 (p-STAT3) exhibiting clear dose-dependent inhibition (Figure 3A). Given the well-established oncogenic role of STAT3 in both hematological and solid tumors, including head and neck cancers, we sought to determine its functional contribution to fedratinib’s activity. Transient transfection with a *STAT3* overexpression plasmid successfully restored STAT3 and p-STAT3 expressions even in the presence of fedratinib (Supplementary Figure S4; Figure 3B). Functionally, STAT3 overexpression not only promoted ESCC cell proliferation and migration but also significantly attenuated the suppressive effects of fedratinib on these processes (Figures 3C,D; Supplementary Figure S5A,B). Moreover, the proapoptotic and G2/M cell cycle-arresting effects of fedratinib were markedly reversed by *STAT3* overexpression (Figures 3E,F; Supplementary Figure S5C,D). Taken together, these results establish the JAK2/STAT3 signaling axis as a critical functional target through which fedratinib inhibits ESCC progression.

### 3.4 Fedratinib modulates the transcription of *vimentin, survivin,* and *cyclin D1*

**FIGURE 4:**
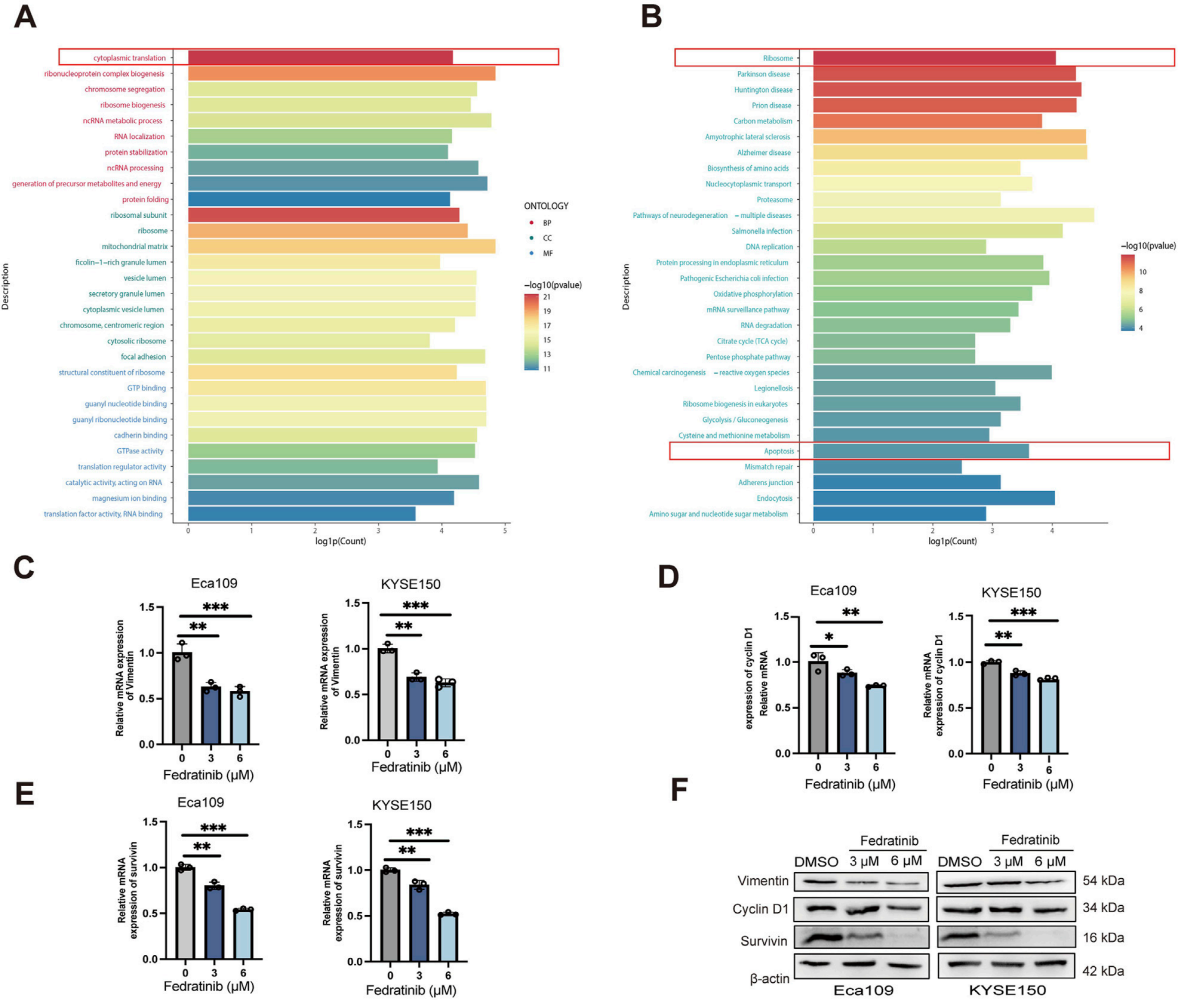
Functional and pathway enrichment analyses of differentially expressed genes (DEGs). **(A)** Gene Ontology (GO) analysis of DEGs. **(B)** Top 30 KEGG pathways ranked by the number of enriched DEGs. **(C–E)** mRNA expression of *vimentin* **(C)**, *cyclin D1* **(D)**, and *survivin* **(E)** in Eca109 and KYSE150 cells treated with fedratinib for 48 h, determined by RT‐qPCR. **(F)** Protein levels of *vimentin, cyclin D1*, and *survivin* detected by Western blot in Eca109 and KYSE150 cells treated with fedratinib (3 or 6 μM) or DMSO. (*p < 0.05, **p < 0.01, ***p <0.001).

To elucidate the molecular mechanisms underlying fedratinib’s antitumor activity, we performed proteomic analysis and identified a set of differentially expressed genes (DEGs). Gene Ontology and pathway enrichment analyses indicated that fedratinib predominantly influences nuclear transcriptional and translational processes (Figures 4A,B). Based on these findings and fedratinib’s known pharmacological profile, we selected vimentin, survivin, and cyclin D1 as potential downstream effectors for further validation. Survivin, a key antiapoptotic protein, is strongly implicated in tumor cell survival and chemoresistance (Siragusa et al., 2024). Vimentin, a type III intermediate filament protein, is a well-established marker of epithelial–mesenchymal transition (EMT) and promotes metastatic behavior by enhancing cellular motility and invasiveness (Tabatabaee et al., 2024). Cyclin D1 plays a central role in driving cell cycle progression, and its overexpression is frequently associated with uncontrolled proliferation and tumor development (Guiley et al., 2019). To evaluate whether fedratinib modulates the expression of these candidates, we treated Eca109 and KYSE150 cells with fedratinib and assessed their mRNA and protein levels. RT-qPCR and Western blot analyses consistently showed that fedratinib significantly downregulated the expressions of *vimentin*, *survivin*, and *cyclin D1* at both transcriptional and translational levels (Figures 4C–F). These results indicate that fedratinib simultaneously targets multiple oncogenic processes—including migration, proliferation, and apoptosis evasion—through coordinated suppression of key effector molecules.

### 3.5 Fedratinib downregulates the expressions of *vimentin*, *survivin*, and *cyclin D1*

**FIGURE 5:**
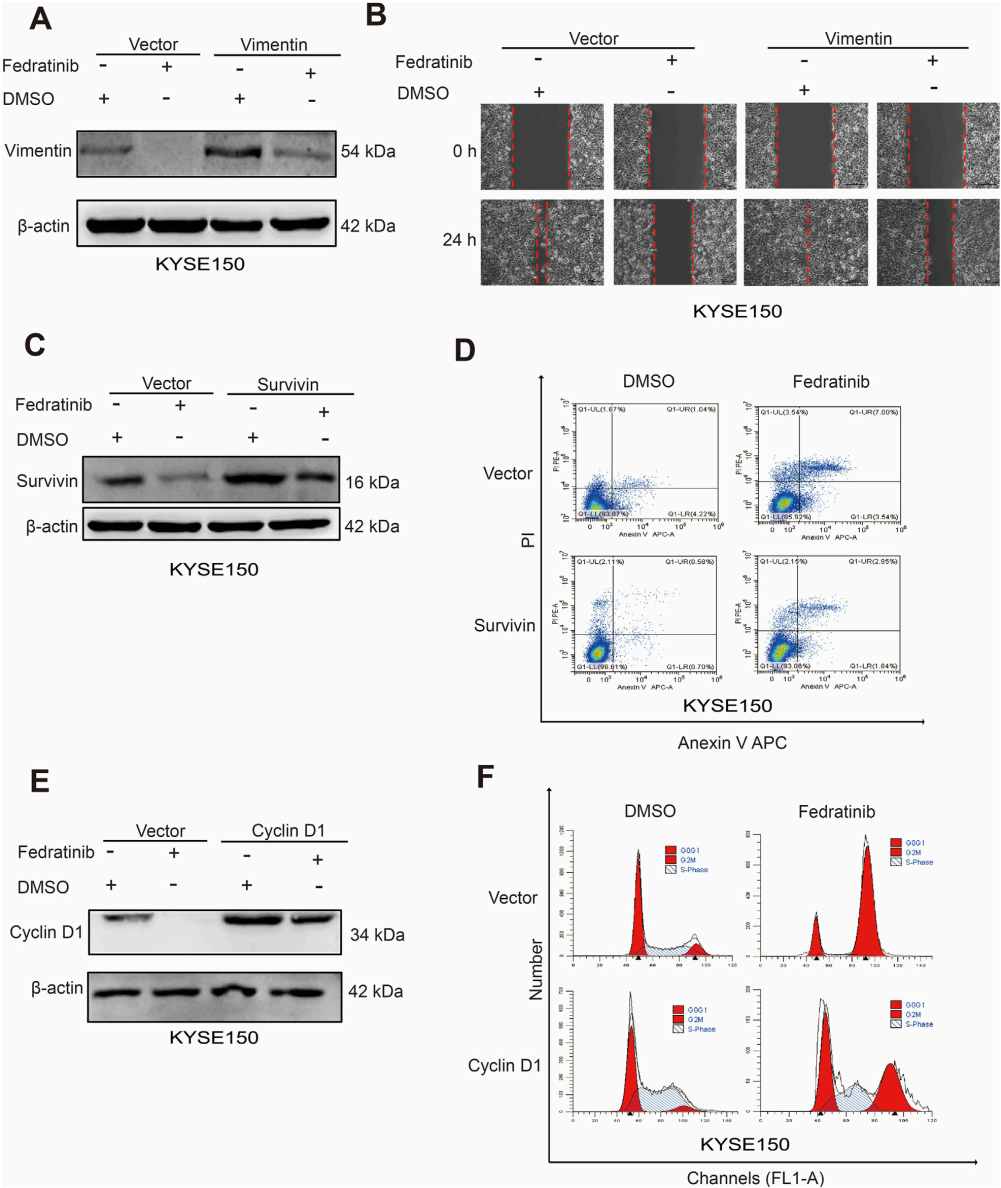
Fedratinib downregulates vimentin, cyclin D1, and survivin expression. **(A)** Vimentin protein levels detected by Western blot in KYSE150 cells overexpressing *vimentin* and treated with fedratinib. **(B)** Effect of *vimentin* overexpression on migration assessed by scratch assay in fedratinib-treated KYSE150 cells. **(C)** Survivin protein levels detected by Western blot in cells overexpressing *survivin* and treated with fedratinib. **(D)** Apoptosis analyzed by flow cytometry in KYSE150 cells overexpressing *survivin* and treated with fedratinib. **(E)** Cyclin D1 protein levels detected by Western blot in cells overexpressing *cyclin D1* and treated with fedratinib. **(F)** Cell cycle distribution analyzed in KYSE150 cells overexpressing *cyclin D1* and treated with fedratinib.

To determine whether *vimentin*, *survivin*, and *cyclin D1* function as downstream effectors of the JAK2/STAT3 pathway in ESCC, we performed plasmid-based overexpression of each gene in fedratinib-treated cells. Western blot analysis showed that ectopic expression of *vimentin* partially reversed fedratinib-induced vimentin downregulation (Figure 5A; Supplementary Figure S6A). Consistent with this result, wound healing assays confirmed that *vimentin* overexpression attenuated the inhibitory effect of fedratinib on ESCC cell migration (Figure 5B; Supplementary Figure S6B). Similarly, *survivin* overexpression restored survivin protein levels and counteracted fedratinib-induced apoptosis (Figures 5C,D; Supplementary Figure S6C,D). Furthermore, *cyclin D1* overexpression partially rescued cyclin D1 expression and alleviated fedratinib-mediated G2/M cell cycle arrest (Figures 5E,F; Supplementary Figure S6E,F). Taken together, these rescue experiments demonstrate that fedratinib suppresses ESCC progression through the JAK2/STAT3 pathway by downregulating *vimentin, cyclin D1*, and *survivin*, leading to concurrent inhibition of proliferation and migration, along with induction of apoptosis.

### 3.6 Fedratinib suppresses tumor growth in ESCC xenograft mouse models

**FIGURE 6:**
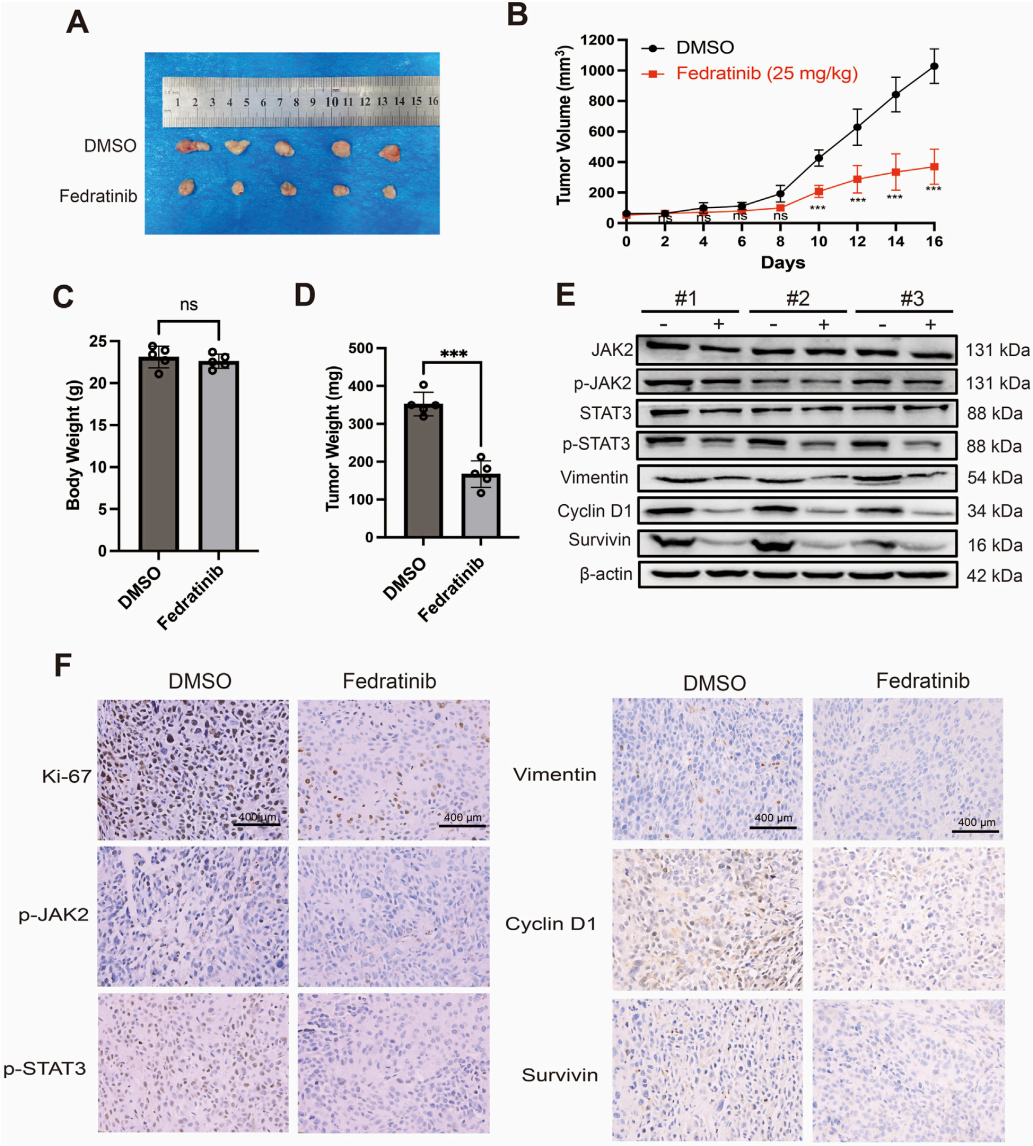
Fedratinib suppresses tumor growth in an ESCC xenograft mouse model. **(A)** Representative images of tumors excised on day 16. **(B)** Tumor growth curves in mice treated with DMSO or fedratinib. **(C)** Body weights of nude mice measured at the endpoint. **(D)** Tumor weights measured after excision. **(E)** Protein levels of JAK2, p-JAK2, STAT3, p-STAT3, vimentin, cyclin D1, and survivin in tumor tissues analyzed using Western blot. **(F)** Immunohistochemical staining of p-JAK2, p-STAT3, vimentin, cyclin D1, and survivin in tumor sections. Data are presented as mean ± SD (n = 5) (ns = no significance, ***p < 0.001).

To evaluate the therapeutic potential of fedratinib *in vivo*, we established subcutaneous xenograft models using KYSE150 cells in nude mice. Intraperitoneal administration of fedratinib (25 mg/kg every 2 days for 16 days) significantly inhibited tumor growth compared to the DMSO control group (Figures 6A,B). No significant reduction in body weight was observed in fedratinib-treated mice relative to controls, suggesting minimal systemic toxicity (Figure 6C). Consistently, tumor weights in the fedratinib group were markedly lower than those in the control group at the endpoint (Figure 6D). Western blot analysis of tumor tissues confirmed that fedratinib downregulated key signaling molecules and effector proteins, including JAK2, p-JAK2, STAT3, p-STAT3, vimentin, survivin, and cyclin D1 (Figure 6E). Immunohistochemical (IHC) staining further demonstrated reduced expressions of Ki-67, p-JAK2, p-STAT3, vimentin, survivin, and cyclin D1 in fedratinib-treated tumors (Figure 6F), indicating suppression of proliferation and progression. Moreover, histopathological examination of major organs (heart, liver, spleen, lung, and kidney) revealed no significant drug-related lesions (Supplementary Figure S7). Taken together, these *in vivo* results demonstrate that fedratinib effectively inhibits ESCC tumor growth without eliciting significant toxicity, supporting its potential as a repurposed therapeutic agent for ESCC.

### 3.7 Fedratinib efficacy in patient-derived ESCC organoids

**FIGURE 7:**
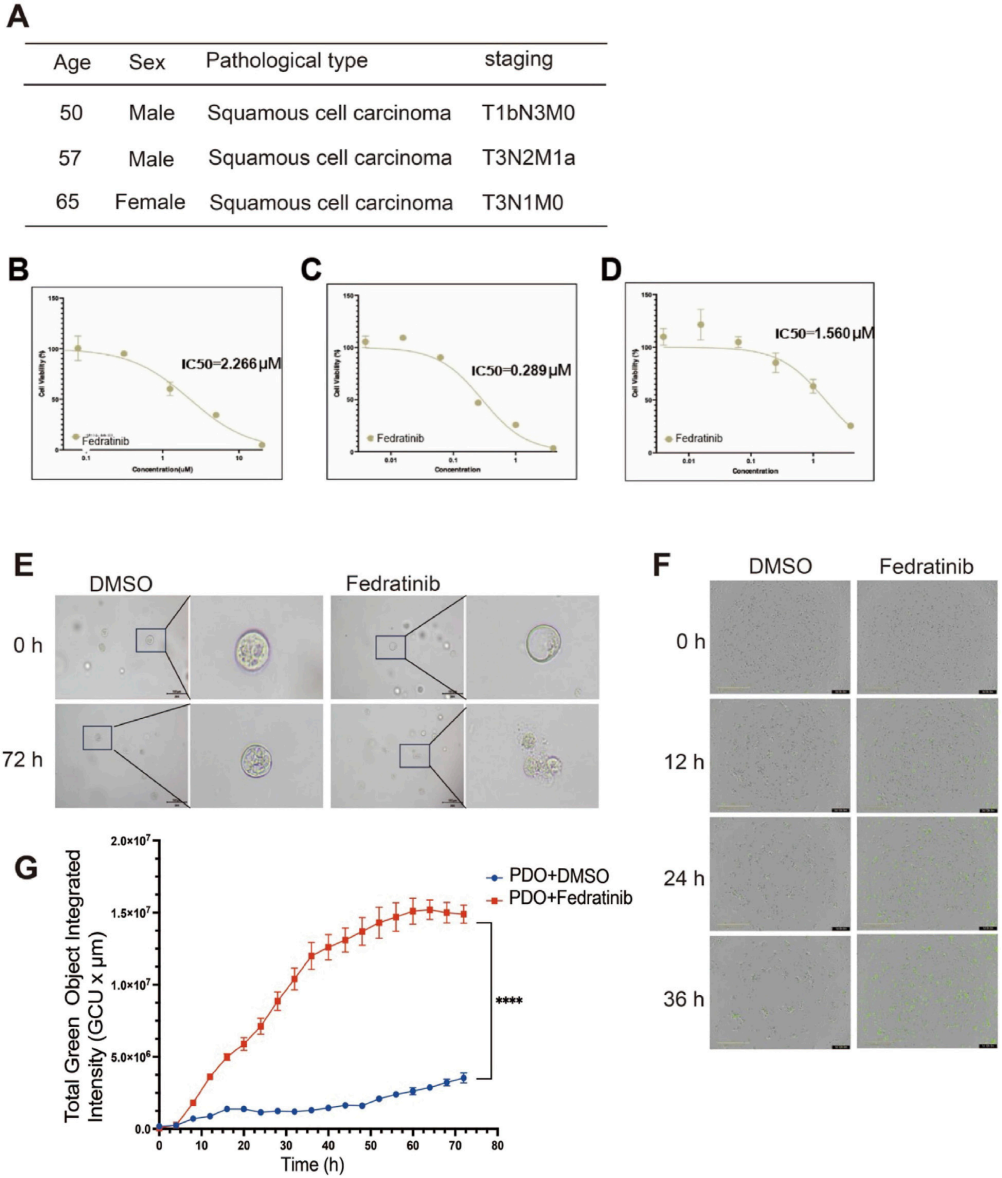
Fedratinib exhibits potent efficacy in patient-derived ESCC organoids. **(A)** Clinical information on the patient from whom organoids were derived. **(B–D)** IC_50_ values of fedratinib in patient-derived ESCC organoids determined using the CellTiter-Glo assay. **(E)** Morphological changes in organoids treated with fedratinib or DMSO. **(F)** Real-time caspase-3/7 fluorescence intensity in organoids treated with fedratinib or DMSO, monitored using an IncuCyte® system. **(G)** Total green object-integrated intensity reflecting apoptosis in organoids. Data are presented as mean ± SD (****p < 0.0001).

Patient-derived tumor organoids have been successfully established for various malignancies, including colorectal, pancreatic, and prostate cancers (Xu et al., 2022). We established PDOs from three patients with pathologically confirmed ESCC, representing a spectrum of clinical backgrounds including varying tumor stages and treatment histories (Supplementary Table S1). To evaluate the efficacy of fedratinib in a clinically relevant model, we generated patient-derived organoids from tumor tissues of three individuals with pathologically confirmed ESCC (Figure 7A). These organoids retained histopathological features of the original tumors upon passaging. Using a CellTiter-Glo Luminescence Assay, we found that fedratinib significantly suppressed the viability of ESCC organoids in a dose-dependent manner (Figures 7B–D). Consistent with this, organoids treated with fedratinib for 72 h exhibited marked morphological disruption compared to the DMSO control group (Figure 7E). To further assess apoptotic response, we monitored caspase-3/7 activation in real time over 72 h using an IncuCyte® Live-Cell Analysis System. Fedratinib treatment significantly increased caspase-3/7 activity (Figure 7F), accompanied by a corresponding decrease in ATP-based cell viability (Figure 7G). Collectively, these results demonstrate the robust antitumor efficacy of fedratinib in patient-derived ESCC organoids, underscoring its potential as a repurposed therapeutic agent. Moreover, the established organoid platform offers a physiologically relevant preclinical model for future drug discovery in ESCC.

## 4 Discussion

**FIGURE 8:**
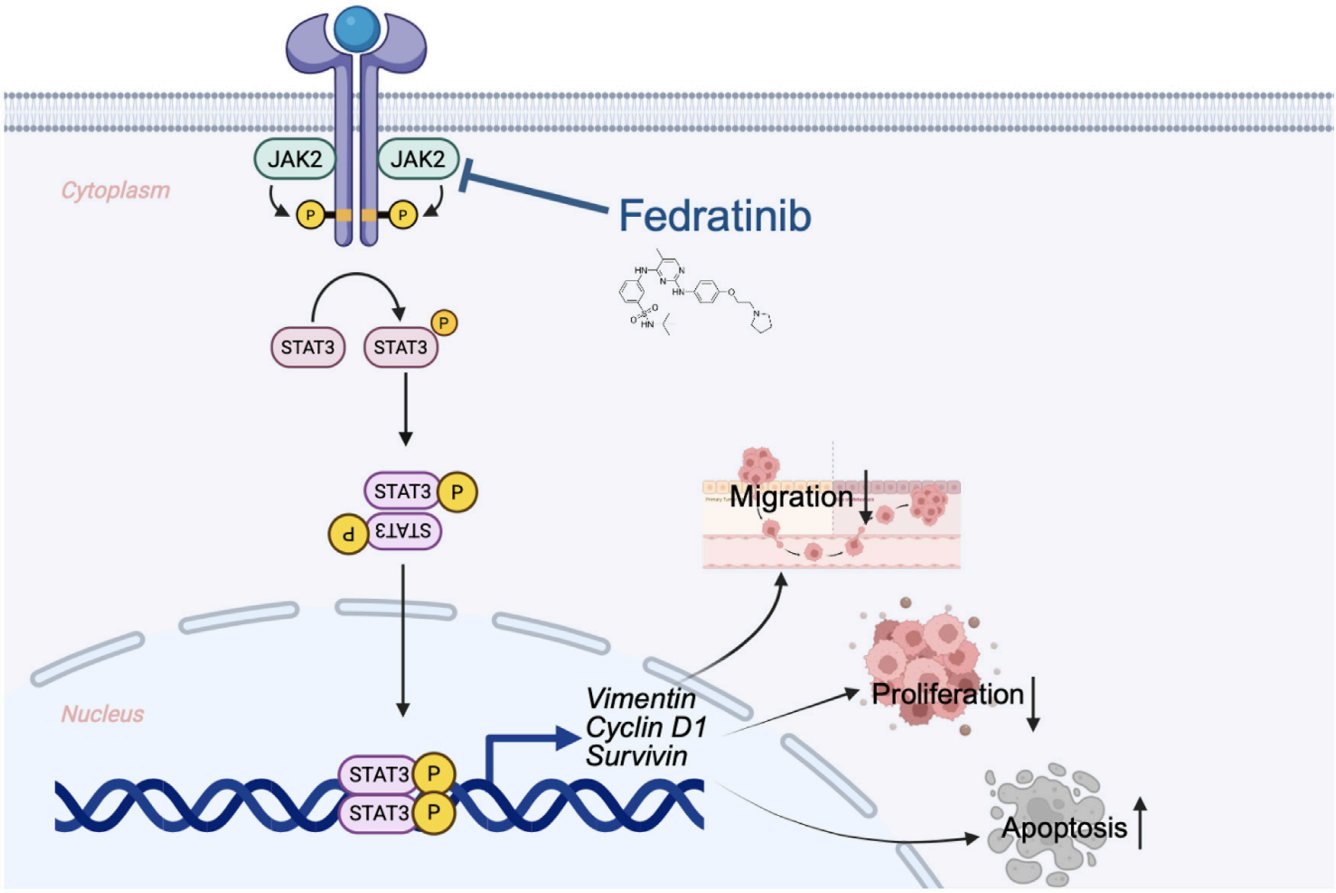
Graphical abstract: molecular mechanism of fedratinib for treating ESCC.

We demonstrated that fedratinib significantly suppresses the proliferation of Eca109 and KYSE150, induces G2/M phase cell cycle arrest, and inhibits ESCC cell migration in a dose-dependent manner. Fedratinib also promoted apoptosis, as evidenced by the upregulation of cleaved caspase-3 and cleaved PARP. Mechanistically, we found that its antitumor activity is mediated through suppression of the JAK2/STAT3 signaling pathway, leading to dose-dependent inhibition of STAT3 phosphorylation and downregulation of key downstream effectors—vimentin, survivin, and cyclin D1 (Figure 8). These results illustrate that fedratinib restrains ESCC progression by targeting JAK2/STAT3 signaling to inhibit proliferation and migration while promoting apoptosis.

Janus kinases (JAKs) are multidomain non-receptor tyrosine kinases that play a critical role in cellular signal transduction. The tyrosine-protein kinase JAK2 mediates intracellular signaling by phosphorylating the transcription factor STAT3, which then translocates into the nucleus and drives the expression of multiple oncogenes (Rah et al., 2022). STAT3 is widely recognized as a proto-oncogene, and its constitutive activation is implicated in the pathogenesis of numerous cancers (Zou et al., 2020). The JAK2/STAT3 signaling pathway regulates a range of key cellular processes, including immune responses, cell proliferation, invasion, migration, apoptosis, and drug resistance (Xiao et al., 2022; Jin et al., 2022; Park et al., 2019). In ESCC, aberrant activation of the JAK/STAT pathway—particularly phospho-STAT3 overexpression—has been frequently observed and is correlated with advanced tumor stage, lymph node migration, and poor prognosis*.* This pathway promotes ESCC progression by enhancing cell survival, facilitating EMT, and contributing to chemotherapy resistance. Given its central role in ESCC pathogenesis, targeting the JAK/STAT signaling axis represents a promising therapeutic strategy for this malignancy. JAK inhibitors are already clinically used in the treatment of myeloproliferative neoplasms, rheumatoid arthritis, and other immune-related disorders (Benucci et al., 2022). In oncology, persistent activation of STAT proteins promotes tumor cell proliferation and survival, fosters a pro-tumor inflammatory microenvironment, and suppresses antitumor immunity (Li et al., 2023). Aberrant activation of the JAK–STAT signaling pathway has been identified in a wide spectrum of cancers, including melanoma, glioblastoma, and carcinomas of the head and neck, lung, pancreas, bladder, breast, colorectal, and prostate (Gomez et al., 2022; Henrik Heiland et al., 2019; Huang et al., 2020; Habanjar et al., 2023). Thus, the repurposing of JAK/STAT inhibitors for ESCC aligns with a growing recognition of their broad antitumor potential.

Drug repurposing has thus gained increasing attention as a promising approach in oncology drug development. Compared with developing new molecular entities, repurposing offers distinct advantages in terms of speed, safety, and cost-effectiveness, thereby overcoming major barriers in conventional drug discovery (Gonzalez-Fierro and Dueñas-González, 2021). Our previous studies indicate that dihydroartemisinin (DHA) and Flexeril have significant anticancer effects on ESCC (Li et al., 2021; Wang et al., 2025; Liu et al., 2025). In this context, the development of selective JAK inhibitors represents a promising therapeutic strategy, with several agents already showing success in early-stage trials. So far, first-generation JAK inhibitors such as ruxolitinib, tofacitinib, and baricitinib have been approved for clinical use in certain cancer settings (Schneider et al., 2022; Bezzio et al., 2023; Kazi et al., 2023).

Previous studies have established that activated (phosphorylated) STAT3 forms dimers and translocates into the nucleus, where it binds to specific response elements in gene promoters and regulates the expression of target genes (Ouyang et al., 2022). In the present study, we demonstrated that fedratinib suppresses proliferation and migration, promotes apoptosis, and inhibits the JAK2/STAT3 signaling pathway in ESCC cells. Dysregulated cell cycle control is a hallmark of tumorigenesis (Matthews et al., 2022), and numerous anticancer agents have been developed to induce sustained G2/M arrest, thereby sensitizing tumor cells to cytotoxic stimuli and ultimately leading to cell death (Chen et al., 2020). Our flow cytometry analysis revealed that fedratinib induces G2/M phase arrest in both Eca109 and KYSE150 cells, and colony formation assays further confirmed its antiproliferative effect. Thus, cell cycle arrest represents one mechanism through which fedratinib inhibits ESCC growth.

STAT3 is widely recognized as a therapeutic target in multiple cancers and has been shown to transcriptionally regulate key effector proteins such as cyclin D1, vimentin, and survivin, which are critically involved in cell proliferation, migration, and apoptosis (Chen et al., 2020; Yang et al., 2019; Kuo et al., 2022). In this study, we demonstrated that fedratinib inhibits STAT3 activity by targeting JAK2 kinase, thereby reducing STAT3 dimerization and nuclear translocation. Although animal models are essential for evaluating *in vivo* drug safety and toxicity, their predictive value for clinical outcomes remains limited (Struzyna and Watt, 2021). An important consideration is how fedratinib’s mechanism of action might differ from that of other JAK–STAT inhibitors. Although first-generation inhibitors such as ruxolitinib target both JAK1 and JAK2, fedratinib is a more selective JAK2 inhibitor with additional activity against FLT3. This distinct kinase inhibition profile may contribute to its potent effects observed in our ESCC models, potentially offering a therapeutic advantage in tumors driven primarily by JAK2 hyperactivation or those with specific dependencies on FLT3 signaling. Furthermore, our data demonstrate that fedratinib effectively suppresses the downstream oncogenic effectors, namely, vimentin, survivin, and cyclin D1, which are critically involved in migration, apoptosis evasion, and proliferation. Although animal models remain essential for evaluating *in vivo* drug safety, their predictive value for clinical outcomes can be limited. Therefore, the consistent efficacy of fedratinib across cell lines, xenografts, and patient-derived organoids in this study strongly underscores its translational potential and suggests that its selective targeting profile warrants further clinical investigation in ESCC.

Patient-derived cancer organoids retain the genetic and phenotypic characteristics of the original tumors and allow for direct correlation with individual patient profiles (Driehuis et al., 2020). In our study, fedratinib significantly suppressed growth and induced apoptosis in ESCC-derived organoids, reinforcing its translational potential. These findings collectively suggest that fedratinib represents a promising repurposed candidate for ESCC treatment. To our knowledge, this is the first study to report the antitumor efficacy of fedratinib in ESCC, providing both mechanistic insights and preclinical evidence to support its further development as a therapeutic option for this aggressive malignancy.

Despite the promising results, our study has several limitations. First, the *in vivo* efficacy of fedratinib was evaluated only in a subcutaneous xenograft model, which may not fully recapitulate the tumor microenvironment or metastatic progression of human ESCC. Second, although patient-derived organoids were used to validate drug response, the sample size was relatively small and derived from a single institution, which may limit the generalizability of the findings. Third, although we identified vimentin, survivin, and cyclin D1 as downstream effectors of the JAK2/STAT3 pathway, other potential targets or compensatory signaling mechanisms remain unexplored. Future studies involving orthotopic or metastatic models, larger patient-derived organoid cohorts, and multi-omics approaches will be necessary to further validate and extend our findings.

## 5 Conclusion

In summary, in this study, we demonstrate that fedratinib suppresses proliferation, migration, and apoptosis resistance in esophageal squamous cell carcinoma through inhibition of the JAK2/STAT3 signaling pathway. Its robust antitumor efficacy was consistently observed across patient-derived organoid models and xenograft mouse experiments, underscoring its translational potential. The concordance of these findings across diverse preclinical platforms provides strong evidence supporting fedratinib as a repurposed therapeutic candidate for ESCC. Our work lays a foundation for the clinical development of fedratinib in ESCC and offers a promising targeted strategy for this disease with limited treatment options.

## Data Availability

The original contributions presented in the study are included in the article/Supplementary Material; further inquiries can be directed to the corresponding authors.
