# Supplementary material for: Fedratinib reveals chemotherapeutic potential in esophageal squamous cell cancer
Source: Front Pharmacol. 2025 Dec 16;16:1689663. doi: 10.3389/fphar.2025.1689663 (PMC12748225; doi:10.3389/fphar.2025.1689663)
Supplement: Supplementary file 1 [file Table1.docx]

**Table S1 Clinical Characteristics of Patients for PDO Generation**

| Clinical Feature | Patient 1 | Patient 2 | Patient 3 |
| --- | --- | --- | --- |
| Chief Complaint | Chest and back pain for >5 months | Dysphagia for >1 month, diagnosed with esophageal cancer for 2 days | Dysphagia for >2 months, diagnosed with esophageal cancer for 5 days |
| Diagnosis | Esophageal mid-segment squamous cell carcinoma | Esophageal lower-segment squamous cell carcinoma, keratinizing | Esophageal lower-segment poorly differentiated squamous cell carcinoma |
| Clinical Stage (cTNM) | T1bN3M0 | T3N2M1a | T3N1M0 |
| Tumor Location | Mid Esophagus | Lower Esophagus (35-40 cm from incisors) | Lower Esophagus (35 cm from incisors) |
| Treatment History (Key Details) | **Neoadjuvant Chemotherapy**  1 cycle of Albumin-paclitaxel + Cisplatin (2023-09-14)  Surgery: Esophagectomy (2023-10-19) | **Treatment-naive**  Admitted for initial treatment planning after recent diagnosis. | **Treatment-naive**  Admitted for initial treatment planning after recent diagnosis. |
| Relevant Biomarkers (IHC) | p53 (strong+), Ki-67 (>60%+) | Not Available | p53 (strong+), Ki-67 (>60%+) |
| Comorbidities | Post-operative complications: esophageal mediastinal fistula, pulmonary infection | Chronic atrophic gastritis | Diabetes Mellitus, Hypertension |
| Smoking History | No | Yes, >40 years, currently smoking | Yes, 43 years, recently quit |
| Alcohol History | No | Yes, >40 years, quit for >1 month | Yes, >40 years, currently drinking |
