# Supplementary material for: Fedratinib reveals chemotherapeutic potential in esophageal squamous cell cancer
Source: Front Pharmacol. 2025 Dec 16;16:1689663. doi: 10.3389/fphar.2025.1689663 (PMC12748225; doi:10.3389/fphar.2025.1689663)
Supplement: Supplementary file 2 [file DataSheet1.pdf]

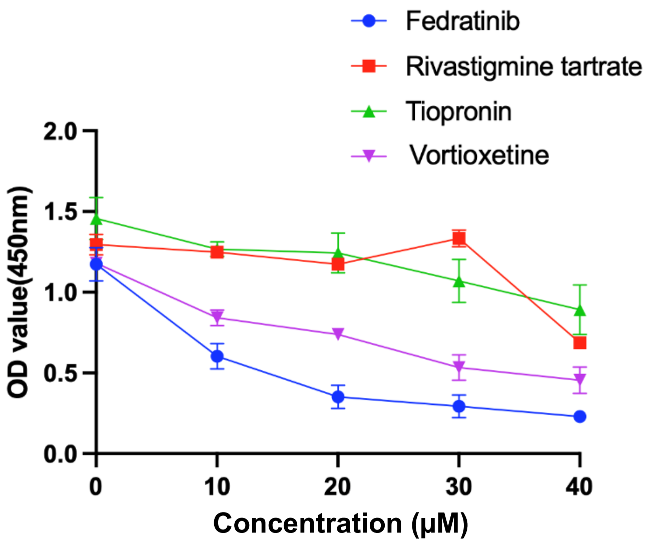

Supplementary Fig. S1 The OD values of primarily selected four drugs were measured at 450 nm.

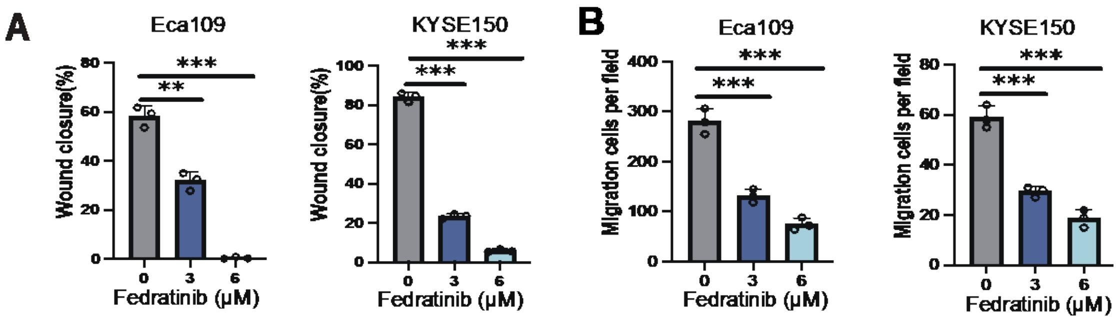

Supplementary Fig. S2 Fedratinib inhibits the metastasis of esophageal cancer cells. Statistical data of Scratch Assay **(A)** and Transwell Assay **(B)** were shown. Student's t-test was used to test differences between two groups. Mean ± S.D. (n = 3) (**p < 0.01, ***p <0.001).

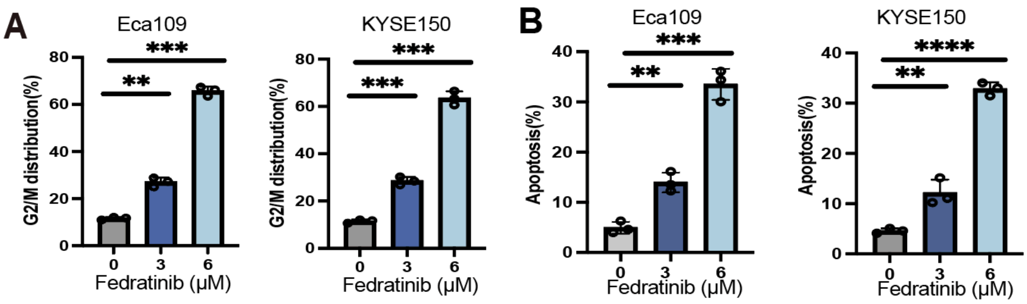

Supplementary Fig. S3 Fedratinib induces cell cycle arrested at G2/M phase and induced cell apoptosis in ESCC cells. Statistical data of cell cycle distribution **(A)** and apoptosis **(B)** of Eca109 and KYSE150 cells treated with fedratinib (3 or 6 μM) or DMSO for 48 hours. Student's t-test was used to test differences between two groups. Mean ± S.D. (n = 3) (**$p < 0.01$, ***$p < 0.001$, ****$p < 0.0001$).

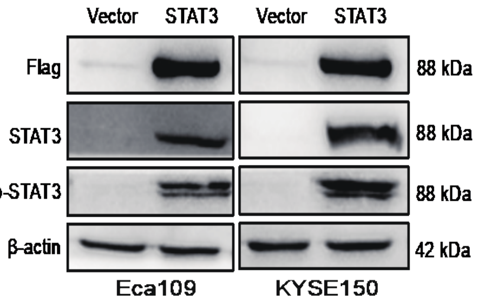

Supplementary Fig. S4 STAT3 plasmids were transient transfectied in Eca109 and KYSE150 cells, deleted by western blot.

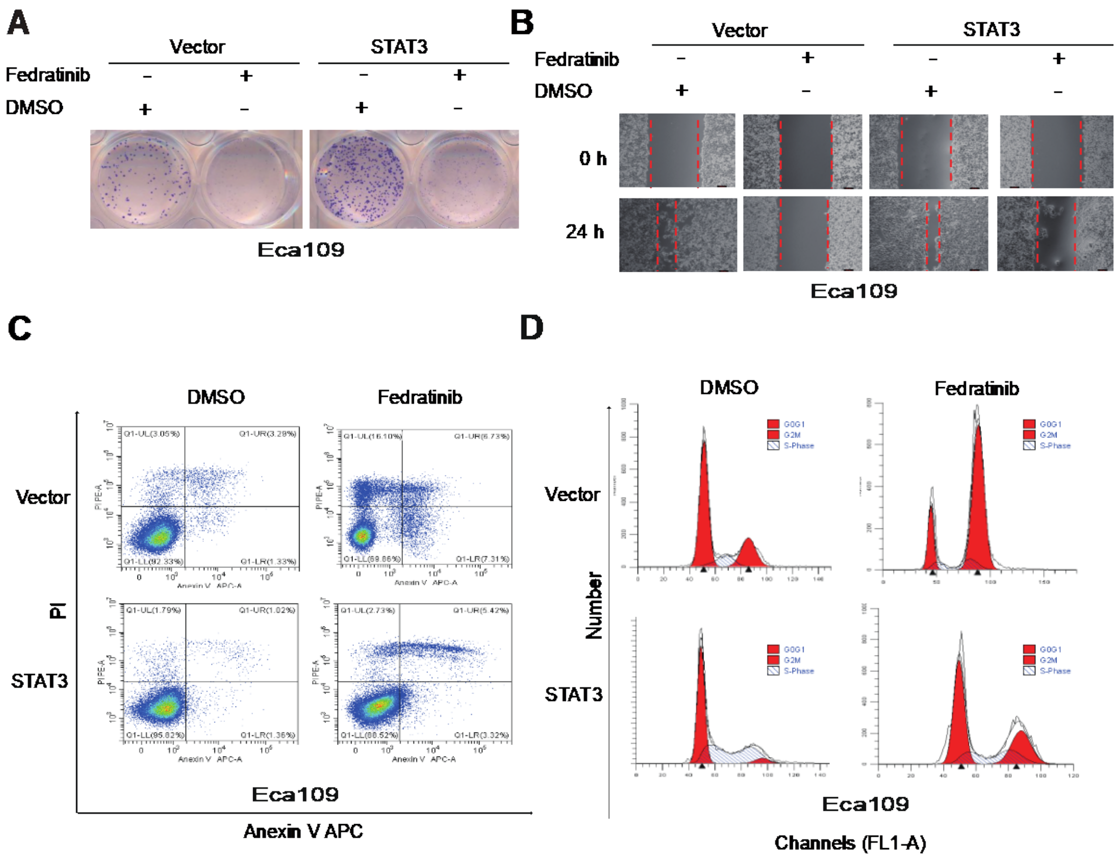

Supplementary Fig. S5 Fedratinib inhibits the JAK2/STAT3 signaling pathway in ESCC cells. **(A)** The effect of overexpression of STAT3 on the proliferation of Eca109 cells treated with fedratinib and evaluated by plate colony formation assay. Scratch assay **(B)** and flow cytometry assay **(C, D)** were employed to determine the migration, cell cycle distribution and apoptosis of Eca109 cells after overexpressing STAT3 and treated with fedratinib.

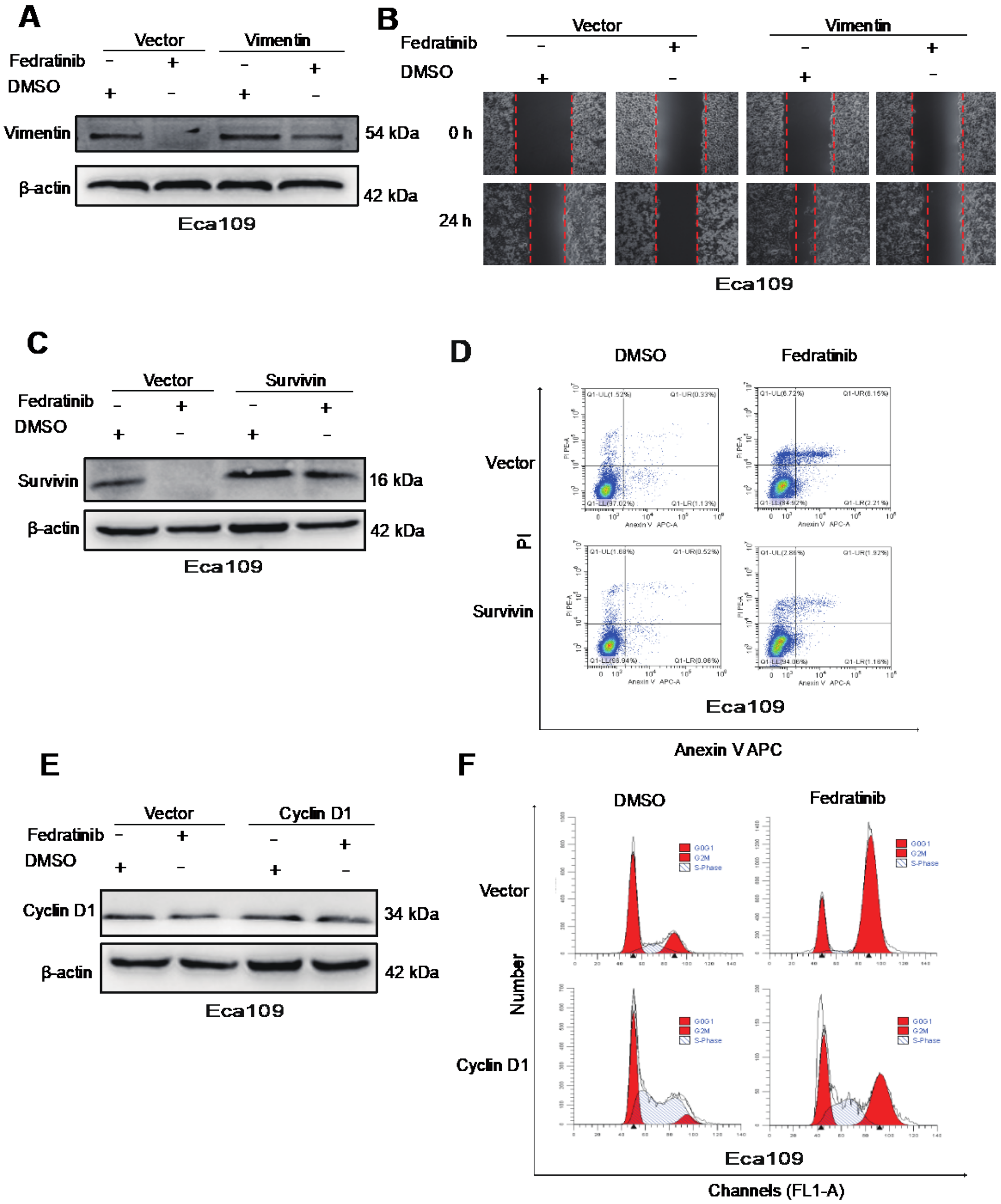

Supplementary Fig. S6 Fedratinib downregulates the expression of vimentin, cyclin D1 and survivin. **(A)**The protein level of vimentin was detected by Western boltting in Eca109 cells after overexpression of vimentin and fedratinib treatment. **(B)** The effect of overexpression of vimentin on the migration of Eca109 cells treated with fedratinib and evaluated by the scratch assay. **(C)** The protein level of survivin was detected by Western boltting after overexpression of survivin and fedratinib treatment. **(D)** Flow cytometry was used to detect the apoptosis-inducing ability after overexpression of survivin and fedratinib treatment in Eca109 cells. **(E)** The protein level of cyclin D1

was detected by Western boltting after overexpression of cyclin D1 and fedratinib treatment. **(F)** cell cycle distribution of Eca109 cells after overexpression of cyclin D1 and fedratinib treatment.

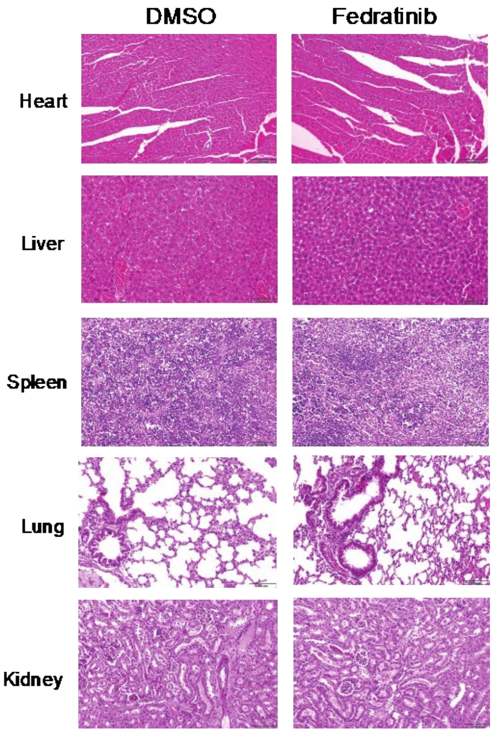

Supplementary Fig. S7 HE staining of heart, liver, spleen, lung, and kidney in xenograft mouse model.
